# Investigation of the Potential Mechanisms Underlying Nuclear F-Actin Organization in Ovarian Cancer Cells by High-Throughput Screening in Combination With Deep Learning

**DOI:** 10.3389/fcell.2022.869531

**Published:** 2022-05-26

**Authors:** Wei Wu, Xiaoxia Xing, Mingyang Wang, Yinzhou Feng, Nina Wietek, Kay Chong, Salma El-Sahhar, Ahmed Ashour Ahmed, Rongyu Zang, Yiyan Zheng

**Affiliations:** ^1^ Department of Gynecologic Oncology, Zhongshan Hospital, Fudan University, Shanghai, China; ^2^ School of Electronic Information and Electrical Engineering, Shanghai Jiao Tong University, Shanghai, China; ^3^ Ovarian Cancer Cell Laboratory, MRC Weatherall Institute of Molecular Medicine, University of Oxford, Oxford, United Kingdom; ^4^ Nuffield Department of Women’s & Reproductive Health, University of Oxford, Oxford, United Kingdom

**Keywords:** nuclear F-actin, ovarian cancer, kinase, high-throughput screening, deep learning

## Abstract

Increasing evidence supports the notion that filamentous actin (F-actin) and globular actin exist in the nuclei of somatic cells, and are involved in chromatin remodeling, gene transcription regulation and DNA damage repair. However, the underlying mechanisms of how nuclear F-actin are polymerized in cells remain incompletely understood. Here, we identify potential kinase targets that participate in nuclear F-actin polymerization in ovarian cancer cells using small-molecule inhibitor library screening in combination with a deep learning approach. The analysis of the targets of the inhibitors used in this study suggest that the PI3K-AKT pathway are involved in regulating nuclear F-actin organization in ovarian cancer cells. Our work lays the foundation for uncovering the important roles of nuclear F-actin in the context of ovarian cancer, and for understanding how nuclear F-actin structures are organized.

## Introduction

Actin is one of the most abundant proteins in the cytoplasm of eukaryotic cells. The incorporation of globular actin monomer into polymers, referred to as filamentous actin (F-actin) polymerization, and depolymerization of F-actin are precisely regulated by a variety of actin binding proteins (ABPs), in response to internal or external stimuli (Pollard and Borisy, 2003; Pollard, 2016). The dynamics of F-actin in the cytoplasm play critical roles in a number of physiological events, including cell motility, cell division, and molecular transport. Despite the cytosolic F-actin being extensively studied, the roles of nuclear F-actin have only recently come under investigation.

Previous reports have noted nuclear F-actin organization in somatic cells using live cell imaging probes, which enable researchers to document the structure of the actin cytoskeleton in nuclei (Baarlink et al., 2013; Belin et al., 2015; Baarlink et al., 2017). Nuclear actin probes, especially the nAc (nuclear actin chromobody) probe, have been employed to directly investigate nuclear actin polymerization, and enable detailed analyses of nuclear F-actin architecture in different contexts (Baarlink et al., 2017; Melak et al., 2017). Involvement of nuclear F-actin has been reported in the modulation of gene transcription (Baarlink et al., 2013; Tsopoulidis et al., 2019), viral particle egress during virus infection (Ohkawa and Welch, 2018), and nuclear volume expansion for chromatin decondensation in mitotic exit (Baarlink et al., 2017). Nuclear F-actin also participates in DNA replication (Parisis et al., 2017), and promotes homology-directed DNA repair (HDR) together with motor protein myosins in the context of DNA damage induced by ionizing radiation or neocarzinostatin (NCS) treatment (Caridi et al., 2018; Schrank et al., 2018). However, whether these nuclear F-actin structures are of clinical significance is not yet known.

Ovarian cancer is the most lethal gynecological malignancy. The main treatment for advanced ovarian cancer is surgical cytoreduction followed by chemotherapy (Coleman et al., 2013; Bowtell et al., 2015). The chemotherapy drug of cisplatin or carboplatin, induces DNA damage and results in cell apoptosis in ovarian cancer. Despite an excellent initial response to chemotherapy, a large proportion of ovarian cancer patients are faced with tumour relapse. The underlying mechanisms of how ovarian cancer cells acquire resistance to chemotherapy remain incompletely understood. Given that nuclear F-actin is involved in DNA damage repair (Caridi et al., 2018; Schrank et al., 2018), it is particularly interesting to examine whether these structures play critical roles in ovarian cancer chemotherapy resistance. Before that, it should be the first step to investigate whether nuclear F-actin structures are detectable in ovarian cancer cells, which have not been reported yet.

Interestingly, the cofilin-bound actin filaments, named cofilin/actin rods, which are unable to be stained by phalloidin (Nishida et al., 1987; Matsuzaki et al., 1988; McGough et al., 1997), have been noted in several diseases. Nuclear cofilin/actin rods have been reported in Huntington’s disease and the irregular expression of the huntingtin protein stabilizes these nuclear F-actin structures (Munsie et al., 2011). In addition, the nuclear cofilin/actin rods are involved in a condition known as intranuclear rod myopathy (Domazetovska et al., 2007). These structures made of nuclear rods also exist in adenosquamous carcinoma cells of the axillary sweat glands (Fukuda et al., 1987). More research is needed to address whether the presence of nuclear cofilin/actin rods are universal in diseases, and whether nuclear F-actin structures differ between diseases.

Moreover, the detailed mechanisms of how nuclear F-actin identified by the nAc probe (Plessner et al., 2015; Baarlink et al., 2017) or nuclear cofilin/actin rods are polymerized remain incompletely understood. It has been reported that several actin binding proteins are involved in F-actin polymerization in the nuclei, which include myopodin, supervillin, espins, formin, and the Arp2/3 complex (Wulfkuhle et al., 1999; Weins et al., 2001; Goley et al., 2006; Loomis et al., 2006; Baarlink et al., 2013; Schrank et al., 2018; Tsopoulidis et al., 2019). MICAL2, which is responsible for redox modification of nuclear actin (Hung et al., 2011), regulates F-actin organization in the nuclei (Lundquist et al., 2014). However, how these factors are regulated by upstream signals has remained elusive.

In this study, we reveal that nuclear F-actin structures are present in ovarian cancer cell lines. Importantly, we further detect that nuclear F-actin exists in ovarian cancer cells in tissue sections. In addition, we identify potential kinase pathways involved in nuclear F-actin polymerization in ovarian cancer cells, using a high-throughput screening strategy in combination with a deep learning approach. These data will pave the way for understanding the roles of nuclear F-actin structures in the context of cancer disease.

## Materials and Methods

### Cell Culture and Patient Sample

HEY and OC316 cell lines were obtained from Robert C. Bast lab in MD Anderson Cancer Centre. SKOV3 and OVCA432 cell lines were purchased from ATCC. All cells were cultured in RPMI1640 medium (Invitrogen), supplemented with 10% fetal bovine serum (Gibco), 1% penicillin-streptomycin (Gibco). The cell lines have been tested and confirmed without mycoplasma contamination. Fresh-frozen tumour sample was from the patient, who provided written informed consent for participation in the prospective biomarker validation study Gynecological Oncology Targeted Therapy Study 01 (GO-Target-01), under research ethics approval 11/SC/0014 by the South Central Berkshire Research Ethics Committee.

### Spheroid Culture

10,000 SKOV3-Lifeact-citrine cells in 50 μl RPMI-1640 medium were dropped on the inside of a tissue culture plate lid and were turned upside down to hang the cells to grow. Sterile PBS was added to the plate to prevent evaporation of the cell droplet. 4 days later, the spheroids were fixed by 4% PFA, followed by DAPI. The images were acquired by Zeiss confocal microscopy equipped with a 63x oil objective.

### Plasmid Construction, Lentivirus Packaging, and Transfection

The Actin-Chromobody (ChromoTek) fused with citrine (a fluorescent tag) and the nuclear localization signal (NLS, SV40 large T antigen), was cloned into the lentiviral expression vector pHR-SIN by MluI and NotI restriction enzyme sites, using the in-fusion cloning kit (Takara). This construct of pHR-SIN:nAc-citrine was generated for expression of nuclear actin probe nAc-citrine (nuclear Actin-Chromobody-citrine) in ovarian cancer cell lines. The lentiviral expression construct of pHR-SIN:Lifeact-citrine was a gift from Marco Fritzsche Lab at the University of Oxford. 2 μg pHR-SIN:nAc-citrine or pHR-SIN:Lifeact-citrine plasmids were packaged with 1 μg pVSVG and 1.5 μg delta 8.2 plasmids using Lipofectamine 3000 transfection reagent in HEK293T cells. Two days later, supernatant from the transfected HEK93T plate was filtered by 0.45 μm filters and then used for infecting different ovarian cancer cell lines.

### High-Content Screening

A library comprising 1247 kinase inhibitors was purchased from TargetMol (Boston, MA, United States) and employed to screen the kinases that are involved in nuclear F-actin polymerization in ovarian cancer cells. 10,000 cells were seeded in a 96-well glass-bottom plate and 1 μM each kinase inhibitor was added in OVCA432 cell line stably expressing nAC-Citrine probe (OVCA432-nAC-Citrine). Twelve hours later, the cells were fixed with 4% paraformaldehyde (PFA) and mounted with anti-fade media containing DAPI. The glass-bottom plates were then read by an HCS machine from which we developed a screening condition that can recognize nuclear F-actin and calculate the frequency of nuclear F-actin containing cells within a vision. Those wells that were treated with kinase inhibitors were compared to the baseline wells which were only treated with DMSO to look for the kinase inhibitors that can decrease the frequency of nuclear F-actin in OVCA432-nAc-citrine.

### Immunofluorescence Staining, Nuclear F-Actin Staining, and Confocal Microscopy

40,000 OVCA432-nAc-citrine cells per well were plated in a 96-well glass-bottom plate (Cellvis) and then fixed with 4% paraformaldehyde for 10 min at room temperature in the following day. The cells were permeabilized with 0.1% Triton-X in PBS for 10 min and then incubated with blocking buffer for 30 min, which comprised of 0.1% Triton-X in PBS and 10 mg/ml bovine serum albumin (Sigma). Next, cells were incubated with anti-Lamin A/C (ab108595, Abcam) antibody overnight and then with fluorescent secondary antibody (Alexa-fluor-568, Invitrogen) for 1 h at room temperature in the dark. For PAX8 staining against ovarian cancer patient-derived tumour cells, patient tumour section slides were employed for anti-PAX8 antibody (1F8-3A8, Thermo Fisher Scientific) incubation and then followed by the similar procedures mentioned above but incubated with fluorescent secondary antibody (Alexa-fluor-488, Invitrogen). Images were captured using the Zeiss confocal microscopy equipped with a 63x or 20x objective.

Ovarian cancer cells of OVCA-432-nAc-citrine were seeded at the density of 1 × 10^5^ per well in a 96-well glass-bottom plate (Cellvis) for at least 6 h before fixed with cytoskeleton buffer (10 mM MES, 150 mM NaCl, 5 mM EGTA, 5 mM glucose and 5 mM MgCl2 at pH 6.1) + 0.1%Triton x100 + 0.25% glutaraldehyde. Wash the cells with PBS and treat them with cytoskeleton buffer +0.2% GA for 30 min. After that, wash the cells 4 times with 1 mg/ml NaBH4 in cytoskeleton buffer which was known to be able to reduce the background autofluorescence from aldehyde fixation. Wash the cells three times with PBS and stain the nuclear F-actin with 0.3 μM Rhodamine-Phalloidin overnight at 4°C. The next day, stain the cells with DAPI and mount them with anti-fade mounting medium (Prolong Gold) before subjected for spinning disc confocal microscopy.

### Bulk Next-Generation RNA Sequencing

Total RNA from 6 × 10^5^ OVCA 432 and OVCA 432-nAC-Citrine (each sample contained three biological replicates) were extracted with RNeasy Plus mini kit (Qiagen) and subjected for RNA quality control using Agilent Bioanalyzer 4200 (Agilent technologies). The QCed RNA was then used for library preparation and subsequent sequencing using Illumina HiSeq 4000 machine with paired end 150bp reading strategy (PE150) and data analyses conducted by Shanghai Biochip Co., LTD.

### The Deep Learning Pipeline for Detecting Cells With Nuclear F-Actin

The deep learning model in this study was based on the 5th version of YOLO series and employed to identify total cells or the cells with nuclear F-actin using the improved convolutional neural network. This network structure was composed of three parts: Backbone for extracting the basic feature, Neck for utilizing the feature, and Output for Prediction. The image samples were annotated for nuclear F-actin detection training. During the model training process, the annotated data was divided into training and validation cohorts. The single-label detection was performed twice for total cells and cells with nuclear F-actin, respectively. This can avoid overfitting potentially caused by the difference in data size when predicting the cells with or without nuclear F-actin simultaneously.

### Statistical Analysis

All experiments were repeated at least three times. Experimental data was depicted as mean ± standard deviation. Statistical analyses were conducted using GraphPad Prism software (version 9) and student t-test or one-way ANNOVA was used to compare different experimental groups.

## Results

### Characterization of Nuclear F-Actin Structures in Ovarian Cancer Cells

**FIGURE 1:**
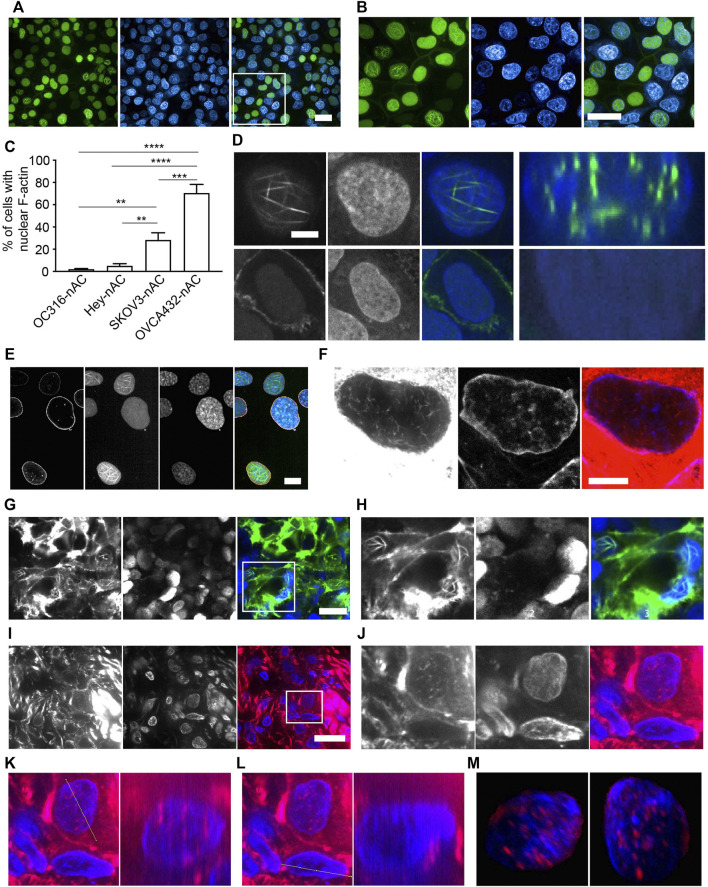
Characterization of nuclear F-actin structure in ovarian cancer cells. **(A-B)**, OVCA432-nAc-citrine cells with nuclear F-actin structures. DAPI, blue. Green, nAc-citrine. A magnified image of the white rectangle is pictured in **(B)**. Scale bar, 20 µm. **(C)**, Proportion of cells with the presence of nuclear F-actin in ovarian cancer cell lines stably expressing nAc-citrine. The statistical analysis was performed using Graphpad Prism 9. The data was shown as mean ± s.e. ** *p* < 0.01 and *** *p* < 0.001.**(D)**, Nuclear F-actin signal (green) within the nucleus of a representative cell of the OVCA432-nAc-citrine cell line obtained using confocal Z-stack scanning and ImageJ Reslice analysis. The bottom shows a cell without nuclear F-actin. Green, nAc-citrine. Blue, DAPI. Scale bar, 5 µm.**(E)**, OVCA432-nAc-citrine cells stained for laminA/C (orange). Green, nAc-citrine. Blue, DAPI. Scale bar, 5 µm. **(F)**, Nuclear F-actin in SKOV3 cells stained using rhodamine-phalloidin. SKOV3 cells were of no nAc-citrine expression. Scale bar, 10 µm. **(G-H)**, Spheroids of SKOV3 cells stably expressing lifeact-citrine imaged after 4 days of spheroid growth. A magnified image of the white rectangle is pictured in **(H)**. Green, lifeact-citrine. Blue, DAPI. Scale bar (in G), 20 µm. **(I-J)**, F-actin staining for a frozen tumour section obtained from a high-grade serous ovarian cancer patient. Red, rhodamine-phalloidin. Blue, DAPI. The white rectangle area was magnified in J, showing a tumour cell stained for nuclear F-actin. Scale bar (in I), 10 µm. **(K-L)**, Reslice algorithm of ImageJ was used to confirm the presence of nuclear F-actin within a patient tumour cell. K, Reslice analysis of a tumour cell (left panel) and rhodamine-phalloidin signal (red) in the nucleus (blue) (right panel). **(L)**, negative control for Reslice analysis (left panel) and a cell without nuclear F-actin due to the absence of rhodamine-phalloidin signal (right panel). **(M)**, Different views of an Imaris-modelled 3D image to visualize nuclear F-actin structures shown in J. Red, rhodamine-phalloidin. Blue, DAPI.

We set out to investigate whether nuclear F-actin is present in cancer cells using ovarian cancer as a model. We generated a variety of ovarian cancer cell lines stably expressing nAc-citrine. nAc (nuclear actin chromobody), a nuclear actin binding probe extensively used to investigate nuclear F-actin (Belin et al., 2015; Plessner et al., 2015; Baarlink et al., 2017; Parisis et al., 2017; Lamm et al., 2020), was fused with the fluorescent tag citrine. Using this probe, we detected nuclear F-actin structures in ovarian cancer cell lines, of which the level of nuclear F-actin varies across different cell lines (Figures 1A–C). Notably, the frequency of nuclear F-actin structures in OVCA432-nAc-citrine cells reaches approximately 70% (Figure 1C). Other cell lines such as HEY-nAc-citrine, rarely displayed F-actin assembly in the nuclei (Figure 1C). The number, thickness, and length of nuclear actin filaments in the ovarian cancer cell line of OVCA432 were further analyzed (Supplementary Figure S1A–C). We obtained Z-stacked image followed by z-slice analysis to show that the strong green signal (nAc-citrine, indicating F-actin binding) was found within the DAPI staining area, confirming the intra-nuclear localization of the nAc-citrine bound F-actin (Figure 1D). Moreover, the lamin A/C staining indicated that F-actin structures did not extend out of the nuclear membrane (Figure 1E). These analyses demonstrated that F-actin assembly occurred in the nuclei of ovarian cancer cell lines. Importantly, the presence of nuclear F-actin was confirmed using phalloidin staining in the SKOV3 cell line in the absence of nAc-citrine expression (Figure 1F), indicating that the nuclear F-actin signals obtained were not an artifact of nAc-citrine tagging and/or ectopic expression. To examine whether the overexpression of nAc-citrine contributes to regulate gene expression in ovarian cancer cell lines, bulk RNA sequencing was performed to compare the transcriptional profiles between OVCA432-nAc-citrine and OVCA432 cells. The saturation analysis, which tells the coverage and depth of the sequencing across samples, showed that the OVCA432-nAc-citrine cells and OVCA432 cells had comparable curves (Supplementary Figure S1D), suggesting that these two cell lines had similar quality of total RNA. Meanwhile, there were very few differentially expressed genes between the cell line of OVCA432-nAc-citrine and OVCA432 (Supplementary Figure S1E), indicating that the overexpression of nAc-citrine unlikely remodels gene expression at a global level. This suggested that it is feasible to document nuclear F-actin structures in ovarian cancer cell lines using the nAc-citrine probe.

To further investigate nuclear F-actin organization in 3D cultures, we generated spheroids of SKOV3 cells, stably expressing lifeact that has been reported as a F-actin binding probe (Riedl et al., 2008) and fused with the fluorescent tag citrine. We detected prominent nuclear F-actin structures in this 3D culture (Figures 1G,H). To further characterize nuclear F-actin organization *de novo*, we performed live cell imaging and recorded F-actin dynamics in the nuclei of SKOV3-nAc-citrine cells, which revealed that the formation of nuclear F-actin structures was not transient (Supplementary Videos S1–S3).

The analysis of nuclear F-actin organization in ovarian cancer cell lines motivated us to check the presence of these structures in tumour cells derived from ovarian cancer patients. The presence of ovarian cancer cells in fresh-frozen tumour sections was confirmed using PAX8 immuno-fluorescence prior to nuclear F-actin staining (Supplementary Figure S2A). The representative images showed that nuclear F-actin was present in tumour cells from a high-grade serous ovarian cancer patient (Figures 1I,J), although these structures were not as organized and striking as those observed in ovarian cancer cell lines (Figures 1A,B). To further confirm the existence of this filamentous organization in the nucleus, we used the same z-slice analysis module of Fiji software and found that some of the red signal (phalloidin binding F-actin) localized in the blue area of DNA staining, suggesting that these F-actin structures were present within the nucleus (Figures 1K,L). To further examine the nuclear F-actin structures in the tumour cell, we employed Imaris to reconstruct a 3D model of the F-actin structures in the nucleus using a series of consecutive images with phalloidin staining, the representative ones of which were shown in Figure 1I,J. It was confirmed again that the F-actin organization was clearly observed within the nucleus (Figure 1M and Supplementary Video S4). Moreover, we noted these nuclear F-actin structures in the cancer cell using another tumour section of the same ovarian cancer patient (Supplementary Figure 2B–E).

### Identifying Kinases that Regulate Nuclear F-Actin Assembly in Ovarian Cancer Cells

**FIGURE 2:**
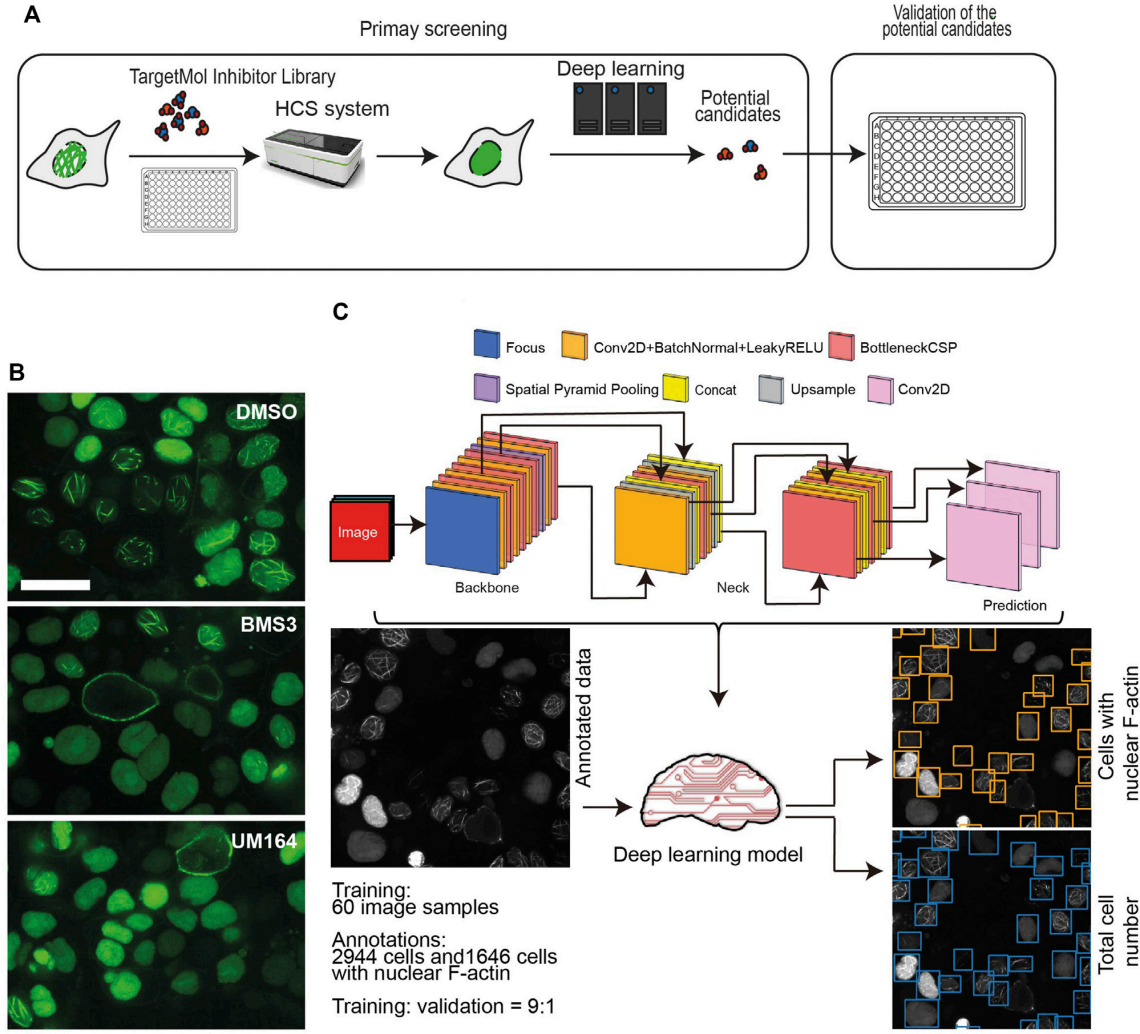
High-throughput screening using a small molecule inhibitor library. **(A)**, A schematic workflow showing the high-throughput screening performed in the OVCA432-nAc-citrine cell line, using TargetMol small molecule inhibitor library and high-content screening followed by a deep learning approach. **(B)**, Representative images of OVCA432-nAc-citrine cells treated with 2.5 µM BMS3 to inhibit LIM kinases, or UM164 to inhibit Src and MAPK kinases. Cells were fixed and stained after 6 h inhibitor treatment. The images were captured using high-content screening. Green, nAc-citrine. Blue, DAPI. Scale bar, 20 µm. **(C)**, The deep learning pipeline is composed of two steps: annotation of nuclear F-actin structures and detection of cells with the presence of nuclear F-actin. The output is shown as cells in which nuclear F-actin structures were detected.

To dissect the mechanisms underlying nuclear F-actin polymerization in ovarian cancer cells, we focused on kinase targets. We were interested in kinases for the following reasons: 1) F-actin structures and dynamics are regulated by a variety of actin-binding proteins (ABPs) (Pollard and Borisy, 2003; Pollard, 2016) , which are subject to various post-translational modifications, including phosphorylation; 2) kinase activity can be directly inhibited by small molecules, which may be repurposed to drugs if kinase-mediated regulation of nuclear F-actin organization proves to be of clinical significance. Thus, we employed the commercial small-molecule inhibitors library TargetMol and High-Content Screening system (HCS) to search for the potential kinase targets that participate in nuclear F-actin organization in ovarian cancer cells. Since the OVCA432-nAc-citrine cell line has prominent F-actin structures in nuclei, we treated these cells with individual small kinase inhibitors from the TargetMol library, which contained 1247 small molecule inhibitors. The screening workflow was shown in Figure 2A.

OVCA432-nAc-citrine were treated with 1 μM individual inhibitor for 12 h, which was of less cytotoxicity to cells during the treatment. The TargetMol small inhibitor library includes a molecule named BMS3, which has been reported to target LIMK kinase (Ross-Macdonald et al., 2008). The previous study has shown that targeting LIMK kinases using the inhibitors, is able to decrease nuclear F-actin levels in U-2OS cells (27). Therefore, BMS3 was employed as a positive control in our high-throughput screening. The images were collected in a high-throughput manner. Briefly, we acquired 30 fields (images) per inhibitor treatment, which produced a substantial set of data of around 38, 000 images in total. The representative images were shown as Figure 2B, indicating that the small molecule of BMS3 and UM164 were able to inhibit nuclear F-actin polymerization in OVCA432-nAc-citrine cells.

**FIGURE 3:**
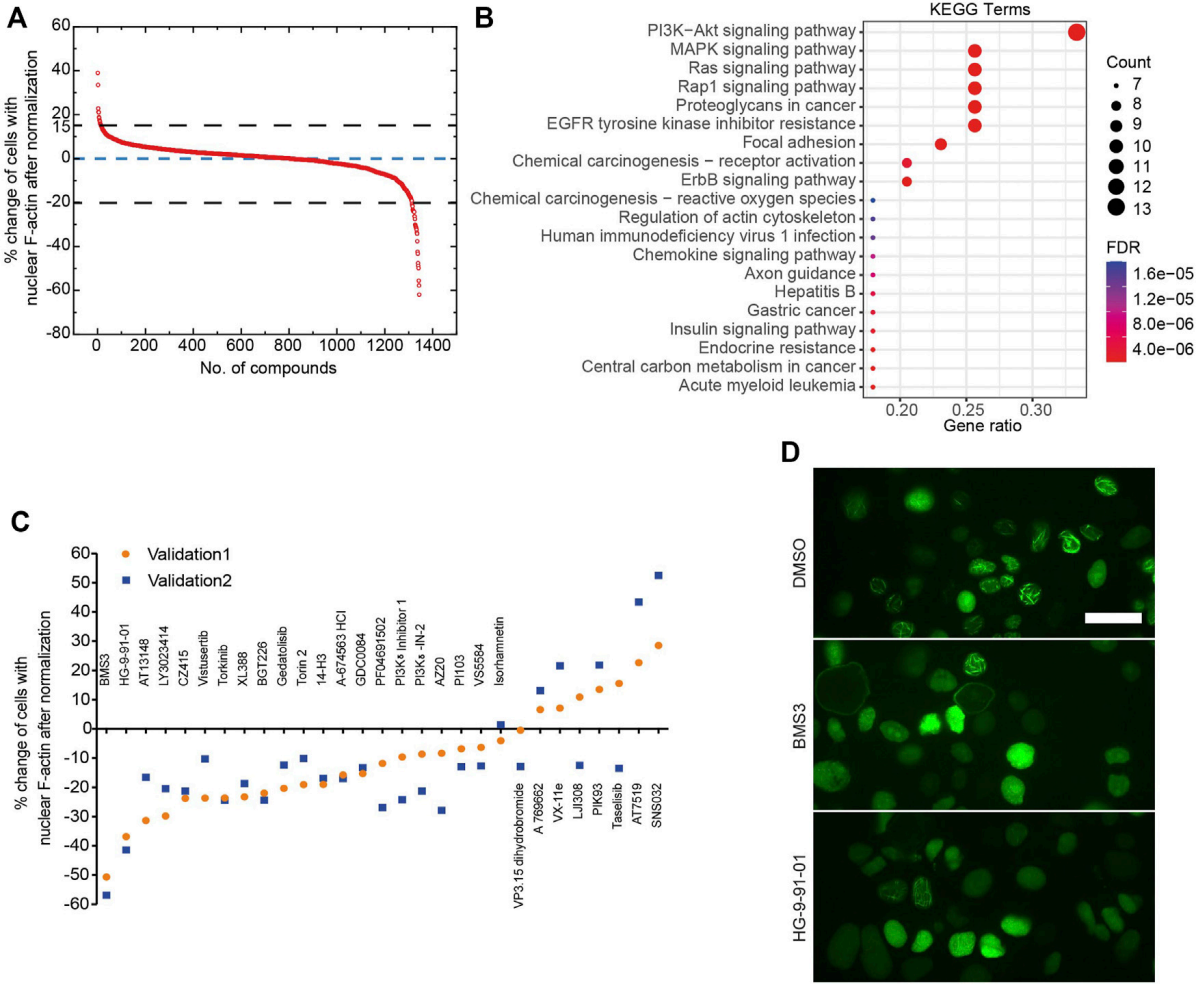
Identification of kinases that are involved in nuclear F-actin assembly in ovarian cancer cells. **(A)**, Summary of the primary screen using 1247 compounds (small molecule inhibitors). The normalized values for OVCA432-nAc-citrine cells treated with individual inhibitors are shown. **(B)**, KEGG analysis of proteins potentially targeted by the compounds, that reduce the percentage of cells with nuclear F-actin by more than 20%, or increase the percentage of cells with nuclear F-actin by more than 15%. **(C)**, The effect of 36 inhibitors targeting PI3K-Akt pathway on nuclear F-actin proportion in OVCA432-nAc-citrine cells. BMS3 was used as a positive control. The orange circles and blue squares represent two biological replicates. **(D)**, Representative images of OVCA432-nAc-citrine cells treated with DMSO, BMS3 or HG-9-91-01. Scale bar, 20 µm.

Next, we developed a deep learning pipeline to profile OVCA432-nAc-citrine cells with nuclear F-actin, using an artificial intelligence framework (Figure 2C). This deep learning model used in this study was based on YOLOv5, which is the latest version of YOLO series (Redmon et al., 2016; Bochkovskiy et al., 2020). In this model, focus layer of the framework is employed to reduce the model’s weights. In the session of training and validating using the annotated cells (Figure 2C), it achieved precision rate of 0.958 and recall rate of 0.891 for total cell detection, and obtained precision rate of 0.917 and recall rate of 0.904 for detection of the cells with nuclear F-actin. Importantly, unannotated cells were randomly assigned for manual counting for total cells or cells with nuclear F-actin, and the result was in agreement with the deep learning model. Thus, this model is a feasible tool to support further image analysis at high-throughput level. Application of this model enabled us to identify the small molecule inhibitors which regulated nuclear F-actin organization in ovarian cancer cells (Figure 3A). We pooled the small molecule inhibitors which reduced the percentage of cells with nuclear F-actin more than 20%, or increased the percentage of cells with nuclear F-actin more than 15%. The kinase targets of these inhibitors were shown in Supplementary Figure S3A,B. We then performed KEGG pathway enrichment analysis using these kinase targets. This identified that PI3K-AKT may participate in nuclear F-actin organization in ovarian cancer cells (Figure 3B). To further confirm the involvement of PI3K-AKT pathway in regulating nuclear F-actin organization, we selected all the kinases that were characterized by the deep learning algorithm due to their effect on remodeling nuclear F-actin structures, 36 inhibitors were shown to be PI3K-AKT pathway related, in which 23 inhibitors had an effect on inhibiting nuclear F-actin polymerization whereas the other 13 inhibitors were shown to promote nuclear F-actin polymerization. We then treated OVCA432-nAc-citrine cells with these 36 inhibitors, together with DMSO as a negative control, and BMS3 as a positive control. The result clearly showed that 21 inhibitors from the down-regulation group, and 4 inhibitors from the up-regulation group had consistent effect on regulating nuclear F-actin organization (Figure 3C,D). Therefore, we were assured that PI3K-AKT pathway does have an important role to play in nuclear F-actin formation.

## Discussion

Most of previous studies investigating nuclear F-actin’s functions have solely been performed using 2D cell culture models. A better characterization of nuclear F-actin architecture in different contexts will ultimately contribute to more detailed analyses of biological roles of nuclear F-actin. Therefore, it is urgent, but also a challenge, to determine the roles of nuclear F-actin at the tissue level, as well as in diseases. In this study, we identified nuclear F-actin structures in ovarian cancer cell lines, spheroid cultures, and fresh-frozen ovarian cancer tumour sections. Importantly, we further revealed that the PI3K-AKT pathway is involved in nuclear F-actin organization in ovarian cancer cells by employing the approaches of high-throughput screening and deep learning. Our research paves the way for further characterization of biological functions and clinical significances of nuclear F-actin structures in the context of cancer diseases.

Despite an initial excellent response to platinum-based chemotherapy in ovarian cancer patients, most suffer tumour relapse within 3 years (du Bois et al., 2003; Ledermann et al., 2013). However, the underlying mechanisms of how this happens are incompletely understood. Previous pioneering studies have demonstrated that nuclear F-actin machinery promotes HDR in fly and mouse cells that have undergone DNA damage caused by ionizing radiation or neocarzinostatin (NCS) treatment (10, 11). But whether this is of clinical significance in ovarian cancer is not yet known. Thus, it would be the key to investigate whether nuclear F-actin polymerization occurs in ovarian cancer cells, which can be useful to uncover the important roles of nuclear F-actin in the context of ovarian cancer.

Our study identified striking nuclear F-actin structures in ovarian cancer cell lines (Figures 1A–C). To our surprise, these filamentous structures have remarkable longevity (Supplementary Videos S1–S3), in contrast to the transient filaments previously reported in other cell types (3–5). It is unlikely that this phenotype in ovarian cancer cell lines is due to an artifact caused by the nAc-citrine probes as: 1) the level of nuclear F-actin structures differs between ovarian cancer cell lines (Figures 1A–C); 2) the nuclear F-actin structure in ovarian cancer cells was confirmed using phalloidin staining (Figures 1A,F toxin which specifically binds to F-actin; 3) bulk RNAseq analysis identified a similar gene expression pattern between the OVCA432-nAc-citrine cell line and the OVCA432 cell line (Supplementary Figure S1D–E), indicating that stable expression of the nAc-citrine probe does not change gene profiling in ovarian cancer cell lines.

Importantly, we identified nuclear F-actin structures in patient-derived ovarian cancer cells (Figures 1I–M). Interestingly, we notice that nuclear F-actin in these cells is not as prominent as the one in culture models of ovarian cancer cell lines (Figure 1 A-B and F). We speculate that this may be due to the limitation of our current nuclear F-actin staining approach using phalloidin as it is a challenge to detect actin filaments in nuclei at the tissue level and it is likely that phalloidin is unable to bind and capture all the nuclear actin filaments at the tumour tissue level. Alternatively, these nuclear F-actin structures, present in the ovarian cancer cells of the fresh-frozen tumour section, may be similar as the nuclear cofilin-actin rods [15–17]. However, this needs to be further confirmed in more tumour sections from more ovarian cancer patients using immuno-fluorescent staining with anti-cofilin antibodies. The limitation of this study is the absence of functional analysis of nuclear F-actin in ovarian cancer cells, which should be conducted in the future. However, the investigation of spatial organization of nuclear F-actin in ovarian cancer cells in this study lays the foundation for further functional analysis of these structures in the context of cancer disease.

Although the existence of nuclear actin and nuclear F-actin has been established here and previous reports, the upstream signaling pathways that regulate nuclear F-actin organization are completely unknown. Our study identified potential kinases, which may be involved in nuclear F-actin organization in ovarian cancer cells. F-actin dynamics (polymerization and depolymerization) are precisely regulated by actin-binding proteins (ABPs) (Pollard, 2016; Pollard and Borisy, 2003). A large number of ABPs have been reported to coordinate F-actin orchestration in the cytoplasm. However, only a few of them have been proclaimed to regulate nuclear F-actin dynamics. In addition, it remains poorly understood whether nuclear ABPs are modified at post-translational level, e.g., by kinase-mediated phosphorylation. Thus, it is necessary to further characterize the roles of ABPs and their potential post-translational modification in tuning F-actin organization in nuclei. In this study, we identified that the PI3K-AKT pathway participates in the regulation of nuclear F-actin polymerization (Figure 3B). To our knowledge, this is the first study to uncover the kinome regulation of nuclear F-actin polymerization at a high-throughput screening level. This work lays the foundation for understanding the detailed roles and potential translational significance of nuclear F-actin in ovarian cancer. However, the detailed mechanisms of how these kinase pathways regulate nuclear F-actin assembly requires further investigation. In the future work, it would be very interesting to test whether kinases from the PI3K-AKT pathway contribute to phosphorylating any nuclear ABPs to orchestrate nuclear F-actin structures in ovarian cancer cells. On the other hand, it has been reported that inhibition of PI3K activity using specific inhibitors accelerated the export of nuclear actin monomer, and thus reduced its concentration in S1 cells (Spencer et al., 2011; Fiore et al., 2017). Since F-actin is assembled by actin monomer incorporation, it would be necessary to examine whether the nuclear F-actin polymerization mediated by PI3K-AKT pathway benefits from maintaining the pool of nuclear actin via PI3K pathway.

Taken together, our study uncovers the presence of F-actin in nuclei of ovarian cancer cell lines, and importantly, reveals the organization of nuclear F-actin in fresh-frozen tumour sections. The first-line treatment for ovarian cancer is cytoreductive surgery in combination with chemotherapeutic agents, including platinum-based drugs. Since platinum-based drugs interlink DNA bases to induce DNA damage in ovarian cancer cells, it would be particularly interesting to test whether nuclear F-actin is involved in DNA damage repair in ovarian cancer cells and whether these structures are of clinical significance in the context of ovarian cancer.

## Data Availability

The datasets presented in this study can be found in online repositories. The names of the repository/repositories and accession number(s) can be found below: https://www.ncbi.nlm.nih.gov/geo/, GSE199915.

## Supplementary Material

Supplementary Figure S1
**(A-C)**, Quantification analysis of nuclear F-actin in OVCA432-nAc-citrine cells. **(A)**,Thickness of nuclear F-actin. **(B)**, Number of nuclear F-actin per cell. **(C)**, Length of nuclear F-actin. **(D)**, Saturation analysis indicating the sequencing coverage. **(E)**, Differentially expressed genes between OVCA432-nAc-citrine cells and OVCA432 cells are shown in volcano plots, in which the significantly up-regulated genes (log2FC>1 and q-value <0.05) are marked in red, and the significantly down-regulated genes (log2FC<-1 and q-value <0.05) are marked in blue.

Supplementary Figure S2
**(A)**, PAX8 stained in a separate frozen tumour section. Green, PAX8. Blue, DAPI. Scale bar, 20 μm. **(B-C)**, Nuclear F-actin staining in a frozen tumour section from the same patient as in Fig.1 I. Red, F-actin staining using rhodamine-phalloidin. Blue, DAPI. The white rectangle was magnified in C. Scale bar (in B), 10 μm. **(D)**, Reslice analysis of a tumour cell (left panel) and rhodamine-phalloidin signal (red) in the nucleus (blue) (right panel). **(E)**, Negative control for Reslice analysis (left panel) and a cell without nuclear F-actin due to the absence of rhodamine-phalloidin signal (right panel).

Supplementary Figure S3
**(A)**, Reported targets of the top 15 small molecule inhibitors that reduced nuclear F-actin polymerization in ovarian cancer cells. **(B)**, Reported targets of the top 5 small molecule inhibitors that increased nuclear F-actin polymerization in ovarian cancer cells.

Supplementary Video S1–S3 Time-lapse movies show representative examples of nuclear F-actin organization of SKOV3-nAc-citrine cells. The movies were generated using images captured by high-content screening. Images were recorded every 5 mins and the movies were displayed with 10 frames per second to show the nucleus F-actin status for approximately 42 h. Green, nAc-citrine.

Supplementary Video S4 Imaris-modelled 3D structure of the nucleus from Figure 1J showing that the actinfilaments localize in the nucleus. Red, rhodamine-phalloidin. Blue, DAPI.
