# Supplementary figures and images for: Investigation of the Potential Mechanisms Underlying Nuclear F-Actin Organization in Ovarian Cancer Cells by High-Throughput Screening in Combination With Deep Learning
Source: Front Cell Dev Biol. 2022 May 26;10:869531. doi: 10.3389/fcell.2022.869531 (PMC9178185; doi:10.3389/fcell.2022.869531)

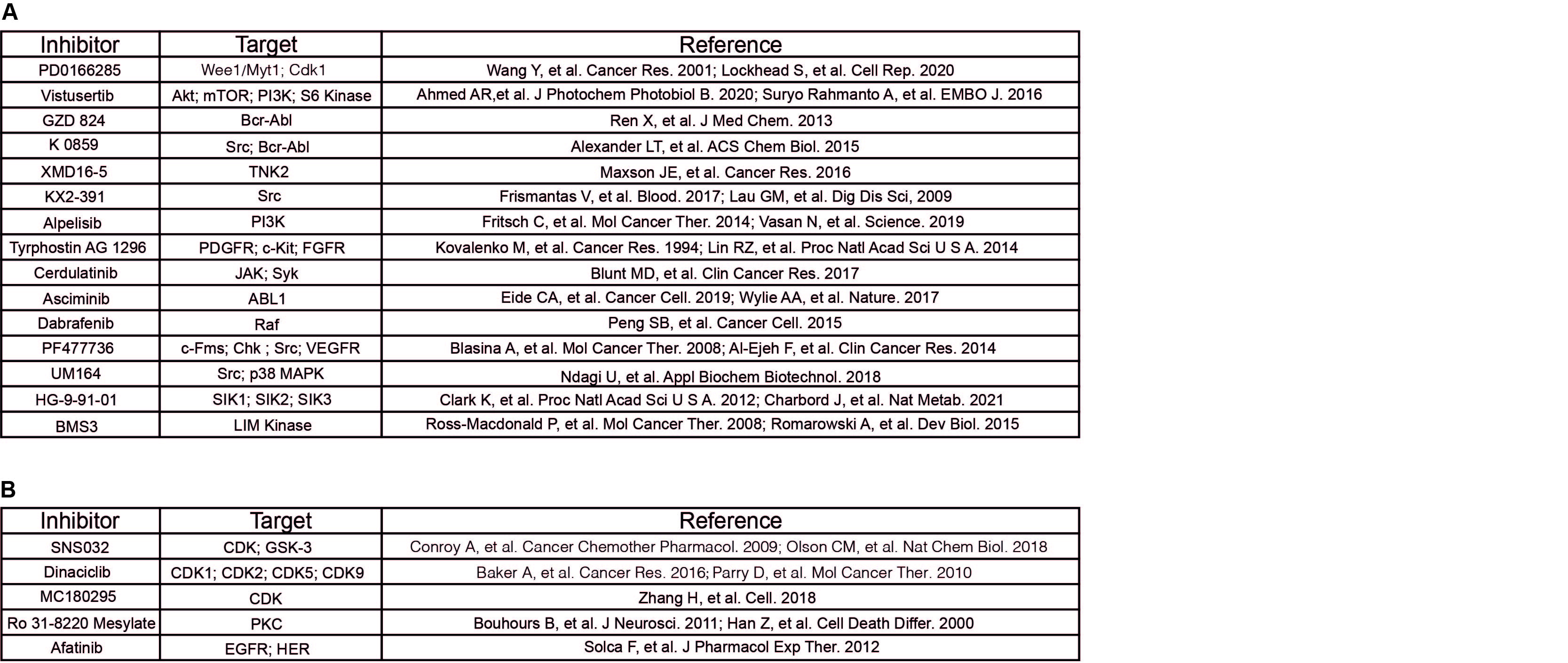

Supplement: Supplementary file 1 [file Image3.JPEG]

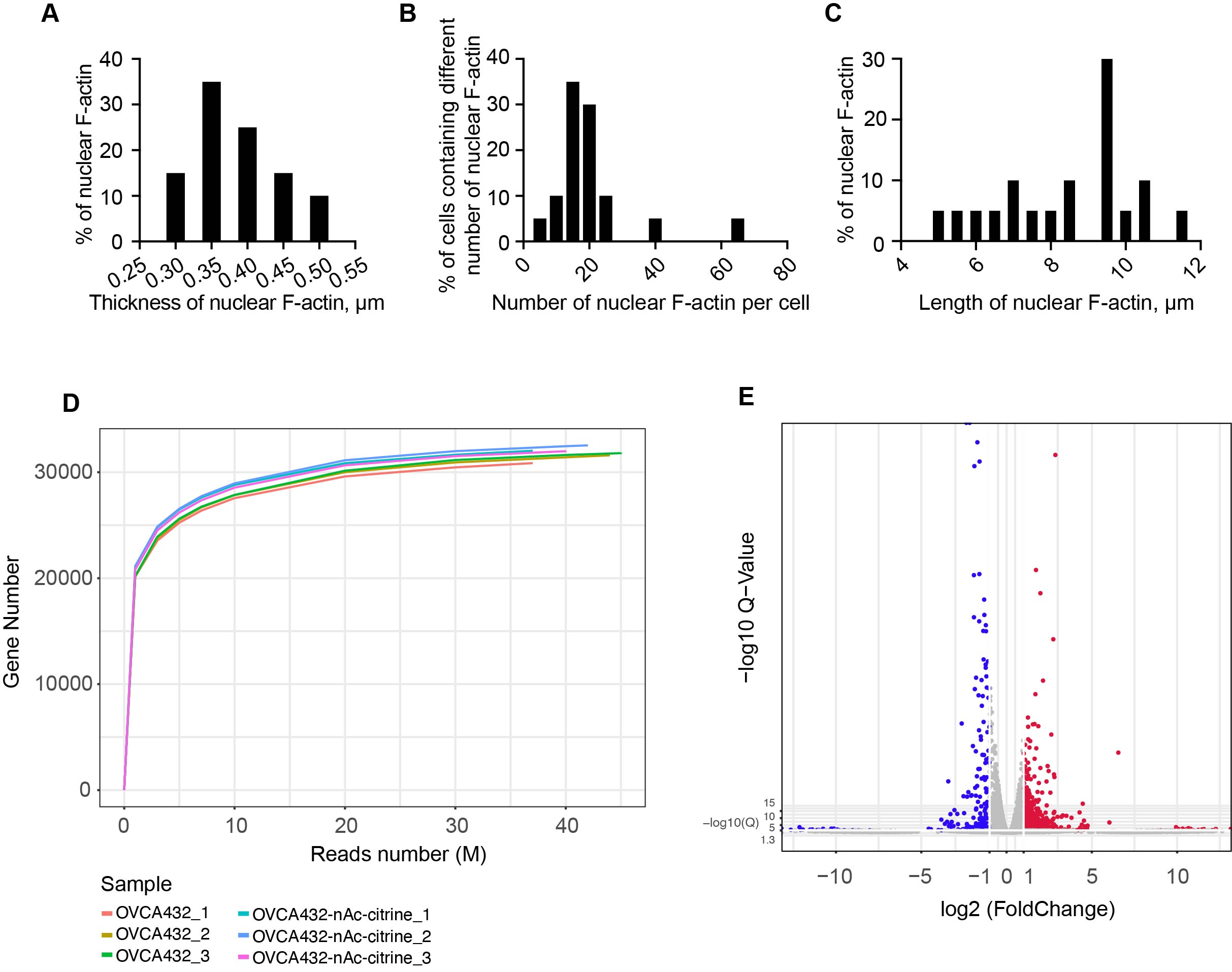

Supplement: Supplementary file 3 [file Image1.JPEG]

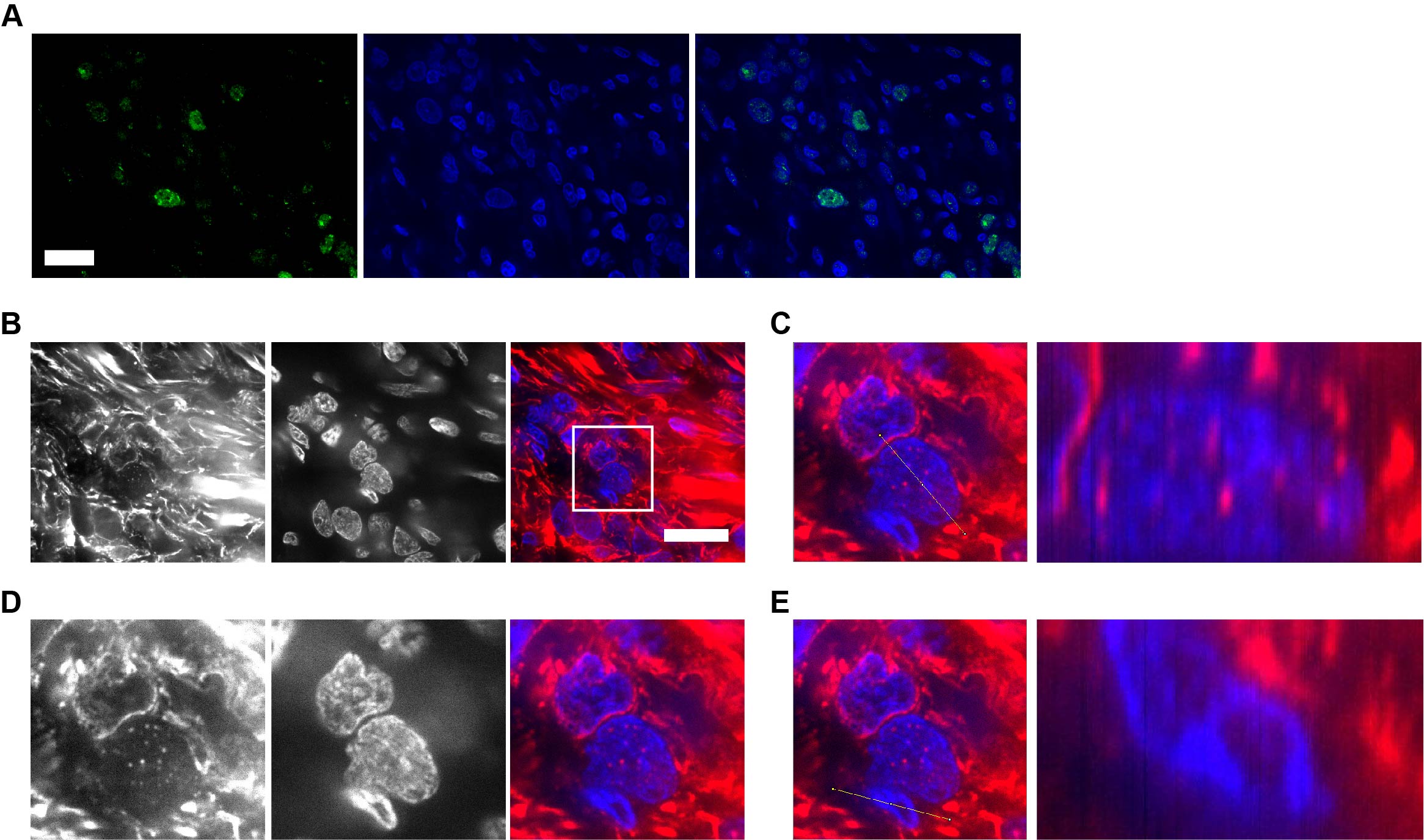

Supplement: Supplementary file 4 [file Image2.JPEG]
